# Surgical Repair of a Coronary Sinus Atrial Septal Defect in an Elderly Patient

**DOI:** 10.1016/j.atssr.2023.11.011

**Published:** 2023-11-28

**Authors:** Fumihiro Miyashita, Tomoaki Suzuki

**Affiliations:** 1 Department of Cardiovascular Surgery, Koto Memorial Hospital, Shiga, Japan; 2 Department of Cardiovascular Surgery, Shiga University of Medical Science, Shiga, Japan

## Abstract

This report describes the case of a 75-year-old man with atrial fibrillation and mitral regurgitation who was diagnosed as having a coronary sinus atrial septal defect, also known as an unroofed coronary sinus, the rarest type of interatrial communication. Elderly patients with this anomaly are sometimes overlooked. However, with the advent of modern imaging techniques, an increasing number of patients with this condition are being diagnosed preoperatively. Our case of coronary sinus atrial septal defect with severe mitral and tricuspid regurgitation, which was treated successfully, is described here and accompanied by detailed surgical images.

Atrial septal defects (ASDs) are congenital heart defects between the atria that cause shunting through the ostium of the coronary sinus (CS) and are stratified into secundum (75% of cases), primum (15%-20%), superior venous defect (5%-10%), and CS septal defect (<1%).^1^ Described also as unroofed CS, coronary sinus atrial septal defect (CSASD) is an uncommon anomaly, often associated with persistent left superior vena cava (PLSVC), and is difficult to diagnose. We have encountered an elderly patient in whom CSASD was diagnosed incidentally at the time of preoperative cardiac computed tomography (CT). This report describes our rare case of CSASD in the absence of PLSVC and includes detailed surgical images.

A 75-year-old man with atrial fibrillation and mitral regurgitation was referred to our hospital with exertional dyspnea. Diuretics had been prescribed by a family physician but failed to improve his symptoms. Electrocardiography showed atrial fibrillation but a stable heart rate of approximately 60 beats/min. A chest radiograph showed cardiomegaly with a cardiothoracic ratio of 70%. Transthoracic echocardiography (TTE) demonstrated severe regurgitation in both the mitral and tricuspid valves and definite dilation of the right atrium, right ventricle, and left atrium (Figure 1). Although the TTE findings indicated severe dilation of the CS, no intracardiac shunt was identified. Cardiac electrocardiographically gated multidetector-row CT confirmed the absence of part of a common wall between the CS and left atrium (Figure 2), which resulted in a diagnosis of CSASD. However, no PLSVC or other congenital cardiovascular anomalies were identified. Cardiac catheterization showed a pulmonary/systemic flow ratio of 2.5.

**Figure 1:**
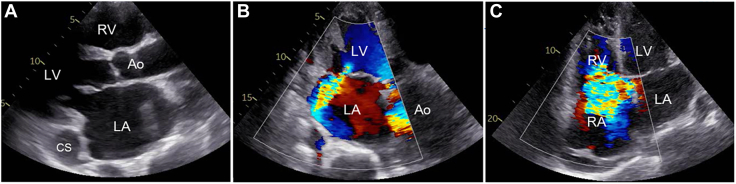
Preoperative transthoracic echocardiography. (A) Parasternal long-axis view shows a dilated left atrium and coronary sinus. (B) Color Doppler echocardiography (3-chamber view) demonstrates severe mitral regurgitation. (C) Color Doppler echocardiography (4-chamber view) demonstrates severe tricuspid regurgitation. (Ao, aorta; CS, coronary sinus; LA, left atrium; LV, left ventricle; RA, right atrium; RV, right ventricle.)

**Figure 2:**
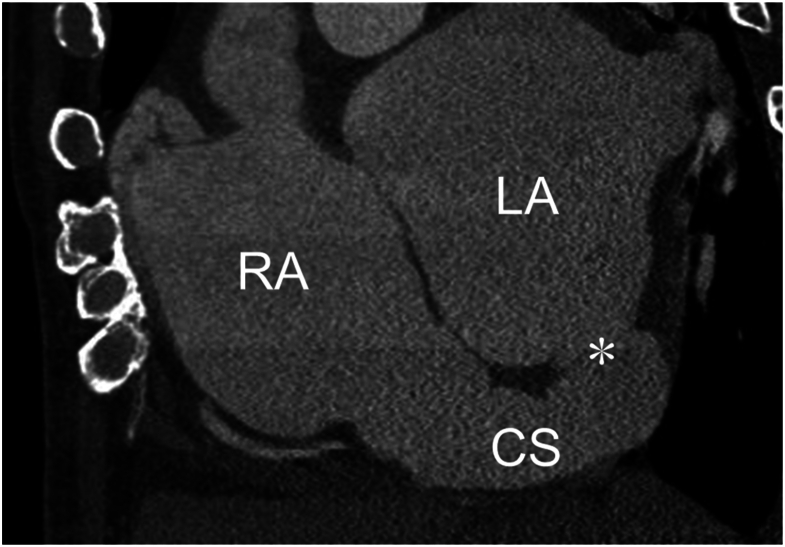
Preoperative cardiac computed tomography scan showing a dilated coronary sinus communicating with the left atrium. ∗Unroofed portion of the coronary sinus. (CS, coronary sinus; LA, left atrium; RA, right atrium.)

Operation was performed through a median sternotomy under standard cardiopulmonary bypass. Aortic cross-clamping and antegrade cardioplegia were performed. The right atrium was opened under cardiac arrest. By a trans-interatrial septal approach, the CS defect was identified near the posterior commissure of the mitral valve (Figure 3A). Given that there was sufficient tissue around the defect, we repaired the CS defect with a direct continuous 4-0 polypropylene suture (Figure 3B). After closure of the defect, cardioplegic solution was administered retrogradely into the CS through the catheter (Video). Dilation of the mitral annulus and mild myxomatous degeneration were observed. Mitral annuloplasty with a 32-mm ring was performed (Figure 3C). Finally, tricuspid annuloplasty was performed with a 31-mm band along with suture annuloplasty (the Kay procedure; Figure 3D). Considering that the left atrium was markedly dilated and anticipating that rhythm control would be difficult, we did not perform a maze procedure. The postoperative course was uncomplicated, and the patient was discharged on postoperative day 15. TTE performed at the time of discharge from the hospital showed trivial mitral and tricuspid regurgitation and no residual interatrial shunting.

**Figure 3:**
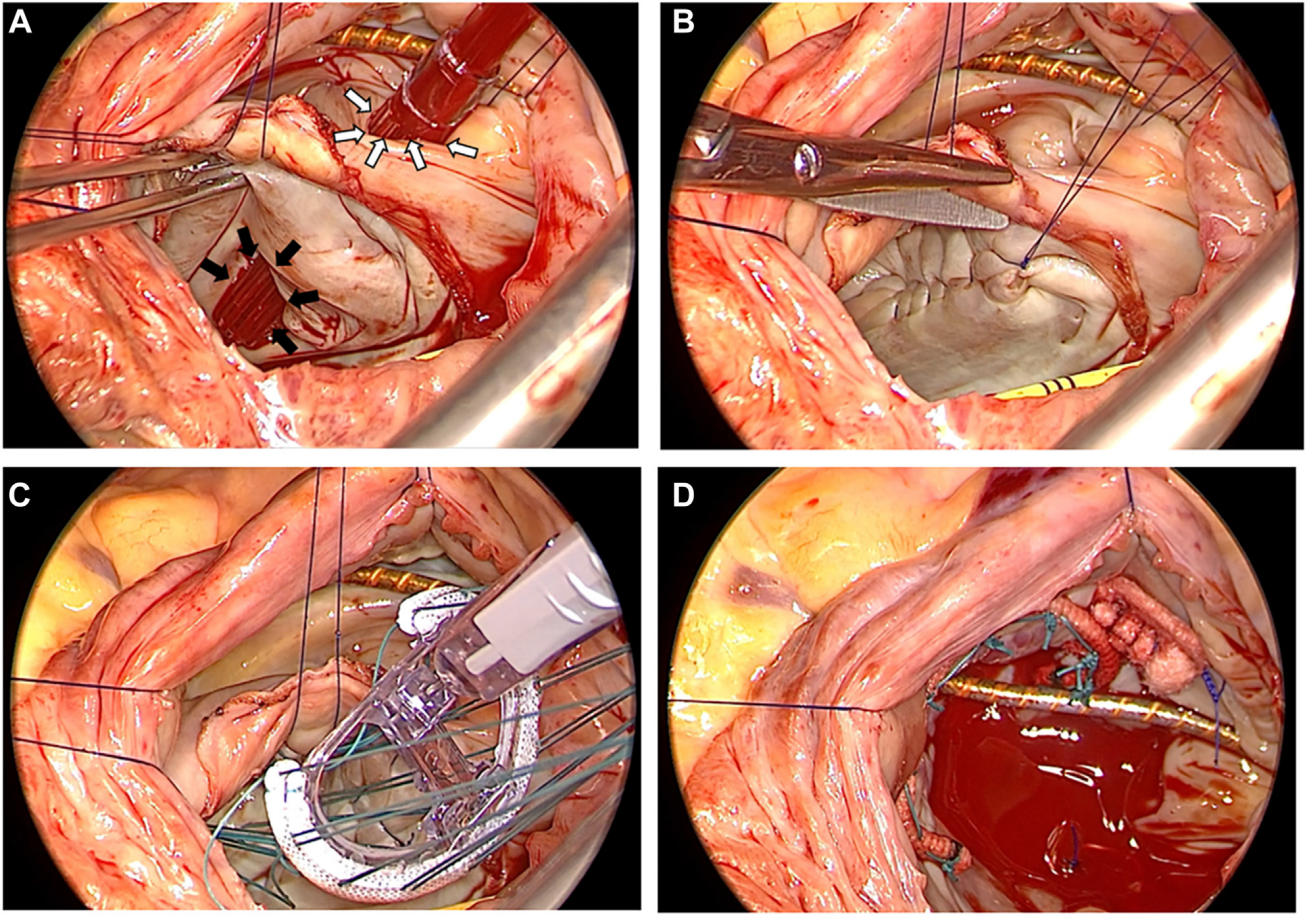
Intraoperative findings. (A) Unroofed portion. We confirmed communication between both atria through the defect (black arrows) by inserting the tip of a cardiac suction tube through the coronary sinus orifice (white arrows). (B) Repair of the coronary sinus with direct continuous suture. (C) Mitral annuloplasty with a 32-mm Physio Flex Ring (Edwards Lifesciences). (D) Tricuspid annuloplasty with a 31-mm Tailor Annuloplasty Band (Abbott Laboratories) along with suture annuloplasty (Kay procedure).

## Comment

CSASD is considered to be a type of unroofed CS. Unroofed CS is a spectrum of cardiac anomalies in which the common wall between the CS and left atrium is absent.^2^ Raghib and coworkers^3^ published the first description of unroofed CS in 1965.

In patients with larger ASDs, long-term volume overload causes atrial and pulmonary enlargement by stretching of the atrial wall and is accompanied by atrial fibrillation, especially in those diagnosed later. In our case, the condition progressed with functional mitral valve regurgitation and secondary tricuspid valve regurgitation, leading to worsening heart failure. Defect closure is safe and effective for improving symptoms, so the surgical indications are the same as those for ASD closure even in elderly patients.^4^ However, in a study that compared the outcomes of early vs late defect closure, Murphy and colleagues^5^ found that age at the time of surgery is the most powerful predictor of long-term survival and that the postoperative complication rate is higher in older patients than in their younger counterparts.^6^ Therefore, early diagnosis and defect closure is desirable to reduce postoperative death and morbidity.

TTE is the noninvasive modality most commonly used in the diagnosis of CSASD. However, cardiac structures that drain to the posterior wall of the left atrium or pulmonary veins are not well visualized by TTE. Moreover, in some cases, transthoracic examination may not be as helpful as it is needed to be because of obesity or scarce transthoracic window. However, cardiac electrocardiographically gated multidetector CT can visualize the exact dimensions of the opening of the unroofed CS and the PLSVC.^7^ Moreover, injection of contrast material into the left arm during the enhanced CT procedure is particularly useful for detection of PLSVC.^7^ Transesophageal echocardiography, cardiovascular magnetic resonance, and cardiac catheterization and angiography are also helpful tools.

In this case, we closed the defect through a median sternotomy because concomitant mitral and tricuspid annuloplasty had been planned before surgery. Although a minimally invasive procedure with or without robotic assistance may have been a reasonable option, there are limitations to such procedures in terms of visualization and an adequate working space. A CSASD can also be repaired percutaneously in cases without PLSVC or valvular regurgitation. However, the safety and efficacy of endovascular procedures have not yet been established because of limited clinical experience.^8^

In conclusion, CSASD is frequently associated with PLSVC and other cardiac anomalies and very rarely encountered in isolation. It is important that CSASD be diagnosed and repaired as early as possible before heart failure worsens. Cardiac CT is useful for diagnosis of not only CSASD but also PLSVC.
